# PG-SGA, GLIM, and CONUT in Hepatopancreatobiliary Cancer: Complementary Tools for Different Clinical Questions

**DOI:** 10.3390/jcm15207883

**Published:** 2026-10-13

**Authors:** Dimitrios Stefanoudakis, Maximos Frountzas, Eva Karaniki, Dimitrios Vouros, Athanasios Emmanouil, Irene Lidoriki, Vasileia Kokala-Dimitropoulou, Dimitrios Theodorou, Dimitrios Schizas, Konstantinos G. Toutouzas

**Affiliations:** 1 First Propaedeutic Department of Surgery, Hippocration General Hospital, School of Medicine, National and Kapodistrian University of Athens, 11527 Athens, Greece; 2 Regional Hepato-Pancreato-Biliary Unit, Manchester Royal Infirmary, Manchester M13 9WL, UK; 3 Department of Environmental, Occupational Medicine and Epidemiology, Harvard T.H. Chan School of Public Health, Boston, MA 02115, USA; 4 Laboratory of Experimental Surgery and Research ‘K. Tountas’, Second Department of Surgery, Aretaieion Hospital, National and Kapodistrian University of Athens, 11528 Athens, Greece; 5 First Department of Surgery, Laikon General Hospital, School of Medicine, National and Kapodistrian University of Athens, 11527 Athens, Greece

**Keywords:** hepatopancreatobiliary cancer, malnutrition, nutritional assessment, PG-SGA, GLIM criteria, CONUT score, sarcopenia, body composition, pancreatic cancer, hepatocellular carcinoma

## Abstract

Background/Objectives: Malnutrition is among the most consequential yet inconsistently addressed comorbidities in hepatopancreatobiliary (HPB) cancer, arising at the intersection of cachexia, sarcopenia, systemic inflammation, hepatic dysfunction, and biliary obstruction. The Patient-Generated Subjective Global Assessment (PG-SGA), the Global Leadership Initiative on Malnutrition (GLIM) framework, and the Controlling Nutritional Status (CONUT) score are often used interchangeably, but they serve different purposes: nutritional assessment, malnutrition diagnosis, and prognostic risk stratification, respectively. This narrative review compares what the three tools measure, summarizes the HPB-specific evidence for each, and proposes a framework for matching the tool to the clinical question. Methods: A structured narrative review was performed. PubMed, Scopus, and Google Scholar were searched for studies applying PG-SGA, GLIM, or CONUT in pancreatic cancer, hepatocellular carcinoma, or biliary tract cancer. HPB-specific evidence was prioritized and the evidence supporting each tool–tumor combination was classified by directness and study design. Results: Applied to the same populations, the tools report different prevalences and classify different patients. In the few HPB cohorts that directly compare tools, PG-SGA classified the largest proportion of patients as malnourished and is the only one of the three designed to record nutrition-impact symptoms and to guide intervention, but it is effort-dependent and may underdetect occult sarcopenia in cirrhosis. GLIM yields a criteria-based diagnosis with severity grading whose prevalence and prognostic value depend on how the muscle mass criterion is operationalized, for example from staging computed tomography. CONUT, computed from three routine laboratory values, is the least resource-intensive and is associated with survival across HPB tumor types in predominantly retrospective cohorts, but its components are confounded by hepatic dysfunction, cholestasis, and inflammation. Conclusions: PG-SGA, GLIM, and CONUT are complementary rather than interchangeable. Because direct comparative evidence in HPB cancer is limited, we propose a framework for matching each tool to the clinical question, tumor subtype, treatment phase and available resources, which requires prospective validation.

## 1. Introduction

Malnutrition is among the most consequential yet inconsistently addressed comorbidities in hepatopancreatobiliary (HPB) cancer. In the landmark Eastern Cooperative Oncology Group analysis, pancreatic and gastric cancers carried the highest prevalence of pre-treatment weight loss [1], and international guidelines now mandate routine nutritional screening in all cancer patients because malnutrition impedes every treatment modality [2]. The burden is amplified in HPB tumors because the affected organs have a central role in digestion, absorption, and metabolism. Cancer cachexia is distinct from sarcopenia and simple malnutrition, and weight- and body mass index (BMI)-based criteria miss muscle depletion in normal-weight or obese patients [3,4].

Each HPB cancer is attributed to a characteristic mechanism. In pancreatic cancer, cachexia affects 30–40% of patients perioperatively and worsens outcomes [5], shortens chemotherapy tolerance [6], and is combined with CT-defined sarcopenia, which independently predicts shorter survival [7,8], with exocrine insufficiency and with biliary obstruction [9]. In hepatocellular carcinoma (HCC), malnutrition is both a cause and a consequence of underlying cirrhosis and substantially affects morbidity and mortality [10], with cachexia and sarcopenia frequently coexisting [11] and sarcopenia worsening survival and complications after hepatectomy [12,13]. In cholangiocarcinoma and gallbladder cancer, a low preoperative Prognostic Nutritional Index predicts worse survival in a meta-analysis [14].

Nutritional assessment is therefore not only descriptive, but prognostic for surgical risk, treatment tolerance, survival, and quality of life, and nutrition is modifiable [15]. Yet the Patient-Generated Subjective Global Assessment (PG-SGA), the Global Leadership Initiative on Malnutrition (GLIM) framework, and the Controlling Nutritional Status (CONUT) score are used interchangeably in HPB oncology, although they represent distinct concepts, particularly because laboratory-based indices are confounded by the inflammation, hepatic dysfunction, and biliary obstruction that characterize HPB disease. This review compares what PG-SGA, GLIM, and CONUT measure, summarizes the HPB-specific evidence for each, and proposes a framework for matching the tool to the clinical question that can be tested prospectively.

This review deliberately focuses on three instruments, each a widely used representative of a distinct class: PG-SGA, a patient- and clinician-completed assessment; GLIM, a consensus-based diagnostic framework; and CONUT, a laboratory-derived prognostic index. Screening tools (NRS-2002, MST, MUST), other laboratory indices (Prognostic Nutritional Index [PNI], Geriatric Nutritional Risk Index [GNRI], C-reactive protein–albumin ratio), and direct body composition measures (CT skeletal muscle index, bioimpedance phase angle) are discussed only where they inform the interpretation or operationalization of the three index tools (Table 1).

## 2. Methods

This is a structured narrative review. PubMed/MEDLINE, Scopus, and Google Scholar were searched from database start to 2 July 2026, combining terms for the target populations (hepatopancreatobiliary, pancreatic, hepatocellular, cholangiocarcinoma, biliary tract, and gallbladder cancer) with terms for the index tools (PG-SGA, GLIM, CONUT and their full names) and for the wider construct (malnutrition, nutritional assessment, nutritional screening, sarcopenia, body composition). The complete search strings were as follows: PubMed/MEDLINE, (hepatopancreatobiliary OR “pancreatic cancer” OR “hepatocellular carcinoma” OR cholangiocarcinoma OR “biliary tract cancer” OR “gallbladder cancer”) AND (“PG-SGA” OR “Patient-Generated Subjective Global Assessment” OR GLIM OR “Global Leadership Initiative on Malnutrition” OR CONUT OR “Controlling Nutritional Status” OR malnutrition OR “nutritional assessment” OR “nutritional screening” OR sarcopenia OR “body composition”); Scopus, (hepatopancreatobiliary OR “pancreatic cancer” OR “hepatocellular carcinoma” OR cholangiocarcinoma OR “biliary tract cancer” OR “gallbladder cancer”) AND (“PG-SGA” OR “Patient-Generated Subjective Global Assessment” OR GLIM OR “Global Leadership Initiative on Malnutrition” OR CONUT OR “Controlling Nutritional Status” OR malnutrition OR “nutritional assessment” OR “nutritional screening” OR sarcopenia OR “body composition”), and Google Scholar, (“pancreatic cancer” OR “hepatocellular carcinoma” OR cholangiocarcinoma OR “biliary tract cancer” OR “gallbladder cancer”) AND (“PG-SGA” OR GLIM OR CONUT).

We included original studies (cohorts, cross-sectional agreement studies, and randomized trials), systematic reviews and meta-analyses, guidelines, and consensus statements that (a) enrolled adults with pancreatic cancer, HCC, cholangiocarcinoma, or gallbladder cancer, or mixed HPB surgical cohorts in which these tumors predominated, and (b) applied PG-SGA, GLIM, or CONUT, or a directly comparable comparator (NRS-2002, SGA, PNI, GNRI, or CT-derived body composition), reporting prevalence, inter-tool agreement, or association with clinical outcomes. We considered studies in non-HPB cancer or non-malignant liver, pancreatic, or biliary disease only when HPB-specific data were absent or when they provided mechanistic evidence on how laboratory markers behave under cholestasis, inflammation, or hepatic dysfunction. Case reports, conference abstracts, pediatric studies, and studies not available in English were excluded. Records were exported to EndNote 20 (version 20; Clarivate Analytics, Philadelphia, PA, USA) and de-duplicated. The PubMed/MEDLINE search identified 1356 records, and Scopus and Google Scholar were searched with equivalent strings to identify additional records. After de-duplication, two authors independently screened titles and abstracts, then full texts, against the eligibility criteria, resolving disagreements by consensus. Finally, the review included 153 sources.

When several studies addressed the same question, evidence was prioritized in the following order: (1) HPB-specific systematic reviews and meta-analyses, (2) HPB-specific prospective or multicenter cohorts, (3) HPB-specific single-center retrospective cohorts, and (4) general oncology or gastrointestinal surgery evidence, cited only where HPB-specific data were lacking. Within each tier, larger, more recent, and methodologically more robust studies were weighted more heavily. For Table 2 the evidence for each tool–tumor combination was classified a priori along two axes: directness (HPB-specific versus extrapolated from non-HPB populations) and study design, adapted from the Oxford Center for Evidence-Based Medicine (OCEBM) 2011 Levels of Evidence for prognostic questions [16]. A GRADE assessment was not undertaken because the questions are descriptive and comparative rather than interventional. Classification was performed independently by two authors, with disagreements resolved by consensus.

## 3. Overlapping but Distinct: Screening, Assessment, Diagnosis, and Prognosis

The nutrition literature often conflates four activities. Nutritional screening is a rapid, usually nurse-administered process that identifies at-risk patients who require further evaluation (e.g., NRS-2002, MST, MUST). Nutritional assessment is a comprehensive evaluation of intake, symptoms, weight history, function, and body composition by a trained professional, designed to characterize the problem and plan intervention (e.g., PG-SGA, SGA). Malnutrition diagnosis applies explicit criteria to classify a patient as malnourished and at what grade severity (GLIM). Prognostic risk stratification uses a marker associated with outcomes, regardless of whether or not the marker is nutritionally specific (CONUT, PNI, GNRI) [49,50]. PG-SGA, GLIM, and CONUT therefore occupy different positions in this sequence (Table 1), and differences in prevalence or prognostic strength among them are expected, not anomalous. Hereafter, we refer to patients as “PG-SGA-assessed,” “GLIM-diagnosed,” or “high-CONUT,” as appropriate.

**Figure 1:**
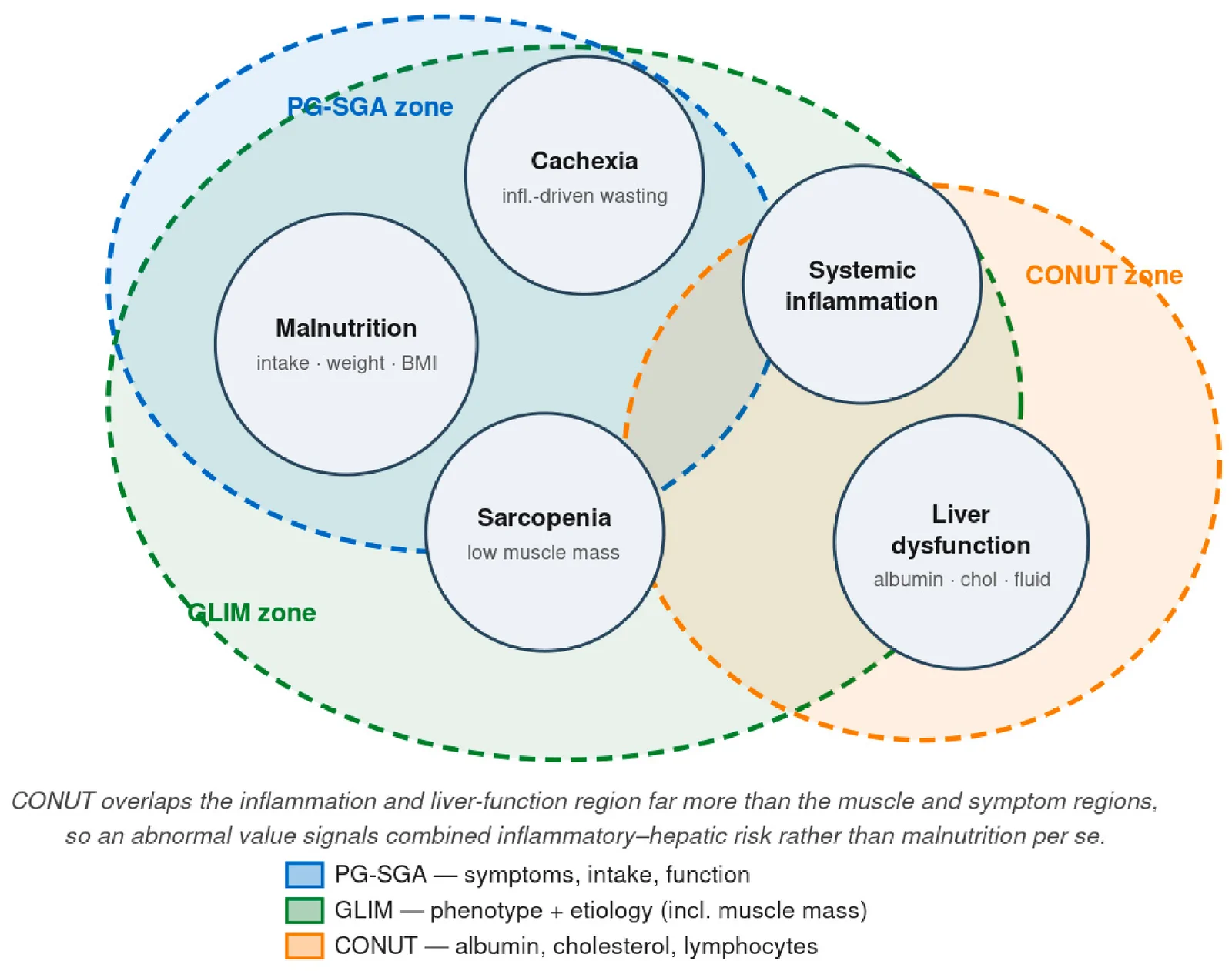
Overlapping but distinct constructs captured by PG-SGA, GLIM, and CONUT. Malnutrition in hepatopancreatobiliary cancer arises from five interrelated domains. Each tool measures a different subset and serves a different purpose: PG-SGA for nutritional assessment and intervention planning, GLIM for criteria-based diagnosis, and CONUT for prognostic risk stratification.

Malnutrition, cachexia, sarcopenia, systemic inflammation, and liver dysfunction are interrelated but not interchangeable in HPB cancer. Patients with cachexia, sarcopenia, or frailty often meet malnutrition criteria. However, nutrition support is less effective when severe inflammation or refractory cachexia predominates [51]. Because PG-SGA, GLIM, and CONUT capture different aspects of this biology, they can legitimately disagree in the same patient (Figure 1).

GLIM operationalizes malnutrition as a two-step diagnosis requiring one phenotypic criterion (weight loss, low BMI, or reduced muscle mass) plus one etiologic criterion (reduced intake or absorption, or inflammation and disease burden) [50]. Cancer cachexia is a narrower, multifactorial, inflammation-driven muscle loss, which cannot be reversed by conventional nutrition [3]. Sarcopenia is defined by low muscle strength confirmed by low muscle quantity or quality [52] and most HPB studies label patients sarcopenic on CT muscle mass alone. Low mass can coexist with normal or high BMI. Muscle quality is a separable, prognostically relevant dimension [53], and body composition changes measurably during systemic therapy in HCC [54].

Another reason the tools diverge is that liver dysfunction, cholestasis, and inflammation distort the laboratory markers on which CONUT depends—albumin, cholesterol, and lymphocyte count—so in HPB disease CONUT may track disease severity as much as nutrition (Section 6). Blood indices and CT muscle metrics carry distinct, non-redundant prognostic information in cholangiocarcinoma [55], so tool selection should follow the clinical question.

## 4. PG-SGA

PG-SGA is the oncology-specific tool of the Subjective Global Assessment [56], adapted by Ottery to include a patient-completed history and triage-to-intervention pathways [57]. It is validated as a scored tool that correlates with quality of life [58]. Patients report weight change, intake, nutrition-impact symptoms and function, while a clinician documents metabolic demand and examines muscle, fat, and fluid. Categories A, B, C, and additive scores (≥4 indicating intervention, ≥9 critical need) triage management [59].

This symptom-aware design suits HPB cancer, where intake is eroded by anorexia, early satiety, nausea, pain, steatorrhea, fatigue, biliary and gastric obstruction, and treatment toxicity. Nutrition-impact symptoms captured by PG-SGA independently predict survival [60], PG-SGA-assessed malnutrition predicts chemotherapy toxicity in other cancers [61,62], and its short form independently predicts survival in large cohorts and correlates with CT-measured muscle loss [63,64]. HPB-specific data are supportive: PG-SGA predicted complications after hepatectomy [28], survival in pancreatic cancer [17], and one-year mortality in HCC [29]. In HPB practice, a score ≥4 has been used to trigger dietitian referral [65], and PG-SGA-assessed malnutrition identified pancreatobiliary surgical patients for preoperative support, improving nutritional indices and quality of life [66].

Its limitations are practical and conceptual. The full tool is time-consuming and requires a trained assessor, whereas the short form reduces the workload but omits the clinician’s examination [63]. It retains subjectivity and depends on patient participation. In cirrhotic HCC, it may underdetect impairment captured by CT muscle and frailty measures [30], and in the available comparative cohorts it flags more patients than GLIM, with agreement ranging from fair in gastric cancer to moderate in HPB surgery [41,67]. Whether this reflects greater sensitivity or over-identification cannot be resolved without a gold standard [68]. HPB-specific validation is uneven. Pancreatic survival data were too sparse to meta-analyze [18], and dedicated biliary studies are essentially absent. PG-SGA can therefore be regarded as a comprehensive assessment and intervention planning instrument downstream of brief screening [69], of particular value where symptom burden and intervention planning dominate, and complementary to—rather than superior to—GLIM or CONUT.

## 5. GLIM

GLIM harmonizes malnutrition diagnosis as a two-step process: first, a positive validated screen, and then confirmation requiring at least one phenotypic criterion (weight loss, low BMI, reduced muscle mass) and one etiologic criterion (reduced intake or absorption, or disease burden and inflammation), with severity graded by phenotype [50]. A 2025 update reaffirmed this architecture [70] and provided guidance on the muscle mass criterion (dual-energy X-ray absorptiometry, CT, or bioimpedance, with anthropometric surrogates where unavailable) [71] and the inflammation criterion [72].

GLIM is attractive in HPB cancer because it separates body composition phenotype from etiologic driver and maps onto cancer-associated malnutrition. In HCC resection, GLIM-diagnosed severe malnutrition predicts shorter overall and recurrence-free survival [73] and contributes independent prognostic information in a multicenter nomogram [31]. In pancreatic and biliary cancer, it predicts major morbidity after pancreaticoduodenectomy [20], failure to achieve a textbook outcome when combined with CT body composition [74], and survival in extrahepatic cholangiocarcinoma [75] and biliary tract cancer on adjuvant chemotherapy [43]. Meta-analyses confirm that GLIM malnutrition worsens survival and increases complications across gastroenterological and broader cancer surgery settings [76,77,78].

Operationalization matters. In one cirrhotic HCC cohort, GLIM prevalence fell from 49.7% to 31.7% when the muscle criterion was omitted, and only the muscle-inclusive diagnosis remained prognostic [32]. In a multicenter colorectal cancer study, muscle mass indicators based on the skeletal muscle index outperformed other surrogates [79]. Yet CT body composition is not universally available, and cutoffs are population-, sex-, and method-specific, with distinct thresholds for Asian gastrointestinal/HPB surgical patients [80] and for cirrhosis [81]. Ascites, edema, and jaundice distort weight, BMI, and muscle measurements, and standard tools—including GLIM—underdetect sarcopenic obesity before pancreaticoduodenectomy [82]. The screening step is not neutral, as many NRS-2002-negative patients are GLIM-malnourished based on CT-derived muscle measurements [83]. GLIM is thus a criteria-based diagnostic backbone whose performance depends on disciplined implementation. It does not automatically outperform PG-SGA or CONUT—in one HPB surgical comparison it was more specific while PG-SGA flagged more patients [41]—and may usefully be combined with symptom-based and laboratory tools, as well as direct CT body composition.

## 6. CONUT

CONUT is an automated index (0–12) derived from serum albumin, total cholesterol, and total lymphocyte count, first validated against subjective and full nutritional assessments in a small general inpatient sample [84]. Its application is simple, inexpensive, objective, and automatable from routine bloods, making it attractive for surgical and oncologic risk stratification, and it predicts survival across surgical oncology [85].

HPB-specific evidence is extensive but almost entirely retrospective. In HCC, high preoperative CONUT predicts poorer survival after resection and under lenvatinib [35,86], post-hepatectomy liver failure [36], and severe early postoperative complications [37]. A meta-analysis confirms this signal across treatment modalities [33] and it complements CT muscle [87,88]. In pancreatic cancer, it independently predicts survival [23], although associations with recurrence and complications are inconsistent and cutoff-dependent [22,24]. In biliary tract cancer, high CONUT predicts worse outcomes but is significantly associated with jaundice itself [44].

These associations must be interpreted in light of persistent confounding, because CONUT’s components are not nutrition-specific. Serum albumin is a negative acute-phase reactant governed by hepatic synthesis, inflammation, and fluid status rather than intake [89]. In cirrhosis, it reflects synthetic failure, driving ascites [90] and edema that in turn distort weight and BMI. Total cholesterol falls with impaired hepatic synthesis and acute illness [91], and lymphocyte count reflects immune reserve and inflammation rather than nutrition [92], also falling with infection and chemotherapy. Inflammation and liver disease therefore reduce albumin and cholesterol independently of intake [93]. Significantly, CONUT also predicts hepatic encephalopathy in non-cancer cirrhosis [94]. Cutoffs range from ≥3 to ≥5, and most of the data are retrospective and from East Asia. CONUT is therefore more appropriately interpreted as a marker of nutritional–inflammatory and physiologic reserve risk rather than a stand-alone malnutrition diagnosis and may be useful for rapid baseline risk stratification and prognostic enrichment, interpreted alongside bilirubin, drainage and infection status, hepatic function, and recent therapy rather than in isolation (Table 3).

## 7. Pancreatic Cancer

Pancreatic cancer presents one of the most complex nutritional profiles in oncology, in which malnutrition reflects the combination of cachexia, anorexia and early satiety, pain, pancreatic exocrine insufficiency with steatorrhea, biliary obstruction, neoadjuvant therapy, surgery, and complications [97]. Pancreatic exocrine insufficiency (PEI) is a guideline-recognized driver of weight loss, steatorrhea, and micronutrient deficiency that none of the three tools captures [9]. Evidence for pancreatic enzyme replacement therapy (PERT) is heterogeneous rather than negative: a small (*n* = 67), 8-week placebo-controlled trial in unresectable disease that did not require documented PEI at enrollment found no significant effect on body weight [98], whereas an earlier randomized trial in unresectable pancreatic head cancer with biliary endoprosthesis showed that PERT prevented weight loss [99], and observational cohorts have associated PERT with longer survival [100,101]. European guidelines therefore recommend PERT when PEI is diagnosed or strongly suspected in pancreatic cancer [2,9,102]. The negative trial reflects sample size, duration, and patient selection rather than evidence against PERT in appropriately diagnosed PEI, which should be assessed and treated separately from the tools reviewed here. A low preoperative L3 skeletal muscle index affects roughly half of surgical patients and independently predicts worse survival [7,8], with longitudinal change more predictive than a single baseline value [103].

Across tools, PG-SGA captures symptom and functional burden, correlates with weight loss, symptoms, and performance status [19], and responds to chemotherapy-based intervention [104]. GLIM provides a standardized diagnosis that agrees substantially with PG-SGA in pancreatic cancer [21] and predicts major morbidity after pancreaticoduodenectomy [20], and its muscle criterion can be read from staging CT. Poor nutritional status and muscle loss also impair the ability to complete adjuvant chemotherapy [105]. CONUT is the most frequently studied laboratory index in pancreatic cancer. In one propensity-matched cohort, it predicted survival independently and outperformed the PNI [23], and it carried the largest pooled hazard ratio among the tools included in a systematic review [18], although that comparison rests on non-overlapping study sets. Its perioperative signal is inconsistent: it predicted survival but not recurrence or complications in one cohort [24], severe complications only at higher cutoffs [25], and pancreatic fistula in another series [26], and it was the weakest complication predictor against the PNI and GNRI in a head-to-head comparison [27]. A meta-analysis confirmed an association with overall survival but not recurrence-free survival [22].

In summary, PG-SGA appears suited to symptom-driven assessment and intervention, although its survival-prognostic value in pancreatic cancer is unconfirmed [18]. GLIM yields a criteria-based diagnosis with severity grading. CONUT, or the similarly albumin-based GNRI [106], is the least resource-intensive prognostic flag and may be used adjunctively. These comparisons rest on limited, retrospective, almost exclusively East Asian head-to-head data with heterogeneous cutoffs, and no tool measures PEI.

## 8. Hepatocellular Carcinoma

HCC is unique because it arises on a cirrhotic, inflamed liver, so the organ determining cancer prognosis also directs the variables used to assess nutrition. Portal hypertension, ascites, edema, inflammation, and sarcopenia make weight, BMI, albumin, cholesterol, and lymphocytes misleading. Sarcopenia affects roughly 38% of cirrhotic patients, scales with Child–Pugh class [107], and predicts worse survival, recurrence, treatment response, and toxicity in HCC, with muscle quality adding further prognostic information [108,109,110]. Because ascites and edema inflate weight and BMI, CT body composition is a more reliable phenotypic anchor.

PG-SGA captures symptoms and function invisible to laboratory scores and is validated and mortality-predictive in cirrhosis [111]. It predicts one-year mortality in HCC [29], and a score ≥4 predicts postoperative complications after hepatectomy [28], although it may underdetect impairment seen on CT in cirrhosis, where muscle- and phase angle-based measures are more sensitive [30,112]. GLIM is applicable in principle, provided that its muscle criterion is operationalized. In cirrhotic HCC, GLIM prevalence and prognostic value depended on including CT muscle, with weight-based GLIM losing its signal [32], while a full GLIM diagnosis predicted survival in a multicenter nomogram [31].

CONUT is prognostic across HCC treatment settings (Section 6). However, a hepatectomy meta-analysis found that CONUT correlated with Child–Pugh class and cirrhosis but not tumor factors, suggesting that it reflects hepatic reserve [34]. Whether CONUT adds value beyond liver function scores remains uncertain. The simpler PNI retained independence where CONUT did not in chemoembolization [38] and outperformed it after resection [39,113]. A cholesterol-modified index outperformed it [114], and after liver transplantation, when the diseased liver had been removed, CONUT was no longer prognostic [40]. Albumin–bilirubin-based scores are themselves robust HCC prognosticators [115,116].

In HCC, therefore, no tool can be read in isolation from liver function. We suggest anchoring diagnosis to GLIM with CT muscle, supplemented by PG-SGA for symptom and functional context and reading CONUT only as an adjunctive prognostic flag whose abnormal values indicate combined hepatic and inflammatory compromise rather than malnutrition.

## 9. Cholangiocarcinoma and Gallbladder Cancer

Biliary tract cancers—cholangiocarcinoma and gallbladder cancer—drive malnutrition through obstructive jaundice and cholestasis, cholangitis, pruritus, anorexia, fat malabsorption, advanced stage at presentation, major surgery, drainage, and chemotherapy. Muscle depletion is common and can be masked by normal or high BMI; so, CT-defined sarcopenia predicts survival despite overweight-range BMI [117], with composite muscle–albumin–inflammation phenotypes showing the strongest signal [118,119,120].

The major problem is that the same obstruction that causes malnutrition misleads the markers used in laboratory scores. The lymphopenia and immune dysfunction of jaundice are endotoxin-driven rather than nutritional [121], albumin remains depressed during obstruction despite adequate intake [122], and superimposed cholangitis adds an acute-phase septic state [123]. Timing matters, since tools yield different results before and after drainage, before surgery, during systemic therapy, and in palliation. Drainage improves symptoms and quality of life [124], and cholangitis and drainage status independently shape morbidity, inflammation, and survival [125,126,127]. Crucially, CT muscle index predicts survival even after percutaneous drainage in jaundiced perihilar disease—precisely where laboratory scores are least reliable [128].

Direct tool-specific evidence is sparse and findings from pancreatic cancer or HCC should not be extrapolated uncritically. PG-SGA captures the symptoms that obstruction produces and, independent of albumin and cholesterol, should in principle be less affected by active obstruction, though dedicated biliary data are limited. GLIM evidence is sparse, but a small cohort showed that severe malnutrition predicted survival [43]. CONUT and related indices predict outcomes across intrahepatic [45,46] and hilar [47] cholangiocarcinoma. High CONUT is significantly associated with jaundice [44] and predicts distant recurrence [48], whereas an explicitly inflammatory C-reactive protein–albumin ratio can outperform it [129]. The simpler Prognostic Nutritional Index predicted survival in a meta-analysis [14] and, combined with the albumin–bilirubin grade, which itself correlates with jaundice, outperformed both in a multi-institutional cohort [130]. Tool interpretation in biliary cancer must therefore account for the timing of obstruction, drainage, infection, and treatment (Table 2).

## 10. Comparative Synthesis

When applied to the same patients, PG-SGA, GLIM, and CONUT report different prevalences, classify different individuals, and predict outcomes with varying strengths, largely because they measure distinct concepts. Direct head-to-head comparisons in HPB cohorts are few [21,27,41,42,131], predominantly retrospective or cross-sectional, mostly from East Asia, and none have compared all three tools against a reference standard in the same HPB cohort. In hepatobiliary pancreatic surgery, malnutrition prevalence was 36.8% by GLIM, 44.6% by PG-SGA, and 60.2% by the biochemical PNI, which agreed poorly with the phenotypic diagnoses [42], and PG-SGA exceeded GLIM in another HPB surgical cohort [41,132,133]. PG-SGA and GLIM agree substantially in pancreatic cancer (kappa 0.71–0.81) [21] but only moderately elsewhere, and agreement shifts with the screening tool used within GLIM [134]. Because no gold standard exists, this discordance is better read as sampling different facets of an unobserved nutritional state than as error [135].

All three predict outcomes, but in different ways. GLIM and PG-SGA both predict complications and survival; in non-HPB cohorts, GLIM was more specific and PG-SGA more sensitive [136], and in one colorectal cohort only PG-SGA predicted long-term survival [137]. CONUT carried the largest pooled hazard ratio among tools in a pancreatic cancer systematic review [18], the albumin-anchored indices (CONUT, PNI, GNRI) behave as a cluster of overlapping inflammatory-prognostic markers [138], yet CONUT is among the weakest classifiers of GLIM-diagnosed malnutrition [95]. Prognostic strength and nutritional specificity are thus separable: a score can predict mortality by capturing tumor burden, inflammation, and organ dysfunction rather than nutritional status.

The tools also differ by design in resource requirements and in what an abnormal result means: CONUT is derived from three routine laboratory values without patient contact but is the least interpretable for nutrition; PG-SGA requires patient participation and a trained assessor but yields an interpretable, intervention-oriented result; GLIM is only as reproducible as its screening step and muscle mass input. Latent-class analyses that assume no gold standard suggest that each tool has a defensible niche rather than that one is correct [139]. The tools are therefore complementary, not interchangeable—PG-SGA is symptom- and intervention-oriented, GLIM is diagnosis-oriented, and CONUT is laboratory-based and prognostic—with a recurring recommendation to screen sensitively and confirm with a standardized framework [131]. The comparative literature does not identify a superior tool. It identifies different tools that answer different questions.

## 11. A Proposed Framework for Tool Selection

**Figure 2:**
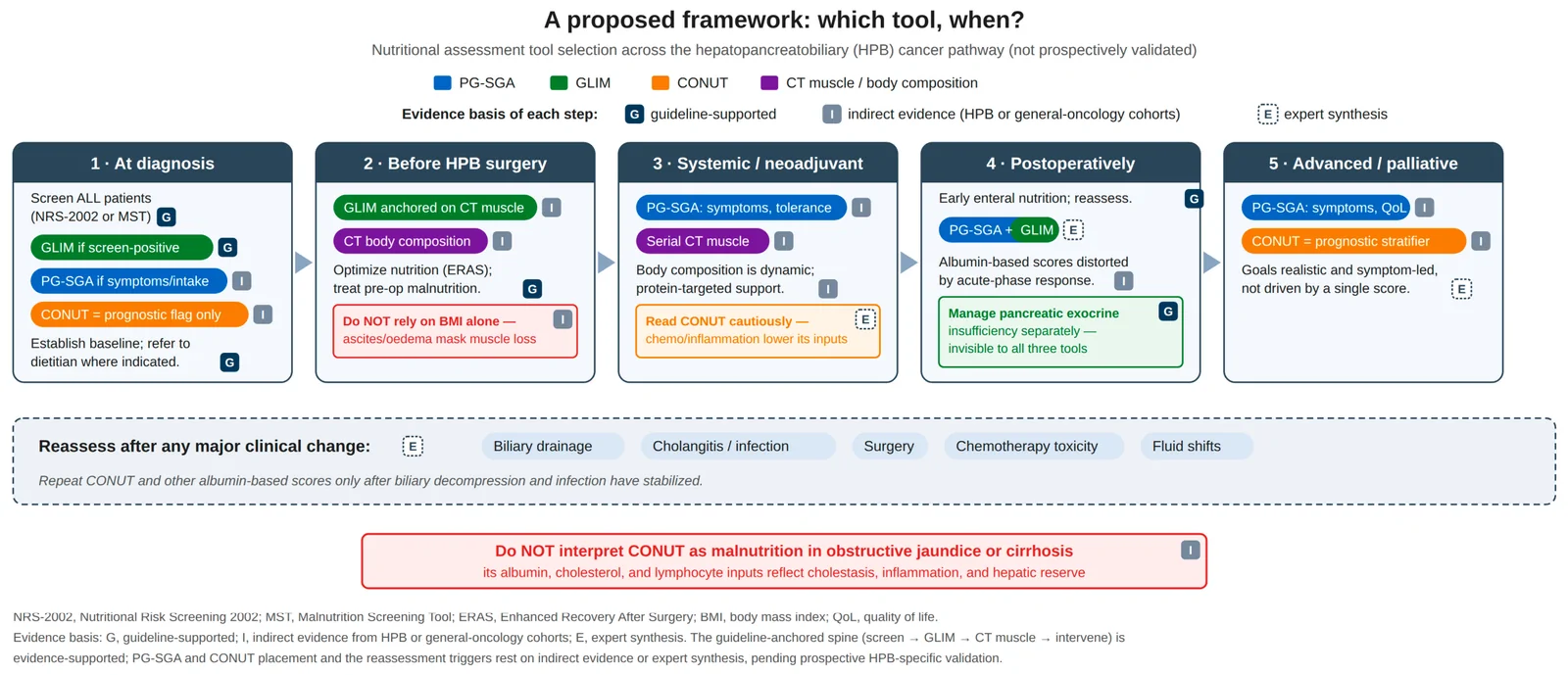
The proposed (non-validated) framework for selecting nutritional assessment tools across the hepatopancreatobiliary (HPB) cancer pathway. Letters indicate the evidence basis of each step: G, guideline-supported; I, indirect evidence; E, expert synthesis.

The framework below (Figure 2) integrates guideline recommendations with indirect evidence and expert synthesis. It has not been prospectively validated and is offered to structure clinical reasoning and to define the comparisons that future studies should test. Each step is tagged G (guideline-supported), I (indirect evidence from HPB or general-oncology cohorts), or E (expert synthesis). The spine of the pathway—screen repeatedly, diagnose and grade with GLIM, use CT muscle where available, and intervene—is guideline-anchored (G): guidelines mandate screening all cancer patients, hospitalized patients, and major-surgery patients, with a validated screen triggering assessment [2,140].

At diagnosis, screen-positive patients should receive a standardized GLIM diagnosis (G) [50], with PG-SGA when symptoms, intake or a dietitian referral predominate (I) [59]. CONUT may serve as an adjunctive prognostic flag (I) [18], not a diagnostic tool, because it classifies GLIM-diagnosed malnutrition poorly [95] and is confounded by HCC and jaundice [141,142]. Before major HPB surgery, preoperative assessment and optimization are guideline-mandated (G) [143,144,145,146], and GLIM may be particularly applicable here because its muscle criterion can use the staging CT that these patients already receive (I) [32,71,147]. During neoadjuvant or systemic therapy, PG-SGA fits the priorities of symptom control and treatment tolerance (I) [59]. Body composition is dynamic and warrants serial CT muscle assessment and protein-targeted support (I) [148,149]. We suggest interpreting CONUT cautiously, because chemotherapy and inflammation depress its components, which the GLIM framework instead treats as an etiologic criterion (E) [150]. Postoperatively, early enteral nutrition and reassessment are standard (G) [143], albumin-based indices are distorted by the acute-phase response, and PEI, invisible to all three scores, should be assessed and treated separately as recommended by guidelines (G) [151]. In advanced or palliative disease, PG-SGA may be appropriate because it directly records symptom and functional burden (I) [18].

Across all phases, we suggest reassessment after major clinical changes, such as biliary drainage, resolution of cholangitis, surgery, toxicity, and fluid shifts (E). This step rests on repeated monitoring and evidence that biliary decompression alters hepatic biochemistry over days to weeks [152,153], implying—though unproven—that CONUT should be re-measured only after decompression and infection have stabilized. A prospective, multicenter HPB cohort applying all three tools and CT body composition at fixed time points, with a pre-specified reference standard and outcome set, would be the appropriate test of this framework.

## 12. Limitations of the Evidence Base and of This Review

Several limitations qualify the comparisons drawn here. First, direct head-to-head comparisons of PG-SGA, GLIM, and CONUT in HPB cancer are few [21,27,41,42,131], predominantly retrospective or cross-sectional, and mostly from East Asia, and no study has compared all three tools against a reference standard in the same HPB cohort. Second, the tools were designed for different purposes, so differences in prevalence or prognostic strength partly reflect construct rather than performance. Third, cutoffs (CONUT ≥3 to ≥5; GLIM muscle mass thresholds) and outcome definitions vary across studies, precluding quantitative synthesis. Finally, the proposed framework (Section 11, Figure 2) integrates guideline recommendations with indirect evidence and expert synthesis and has not been prospectively validated. It is offered as a structure for clinical reasoning and for future comparative studies rather than as a validated algorithm.

## 13. Conclusions

Malnutrition in HPB cancer arises at the intersection of cachexia, sarcopenia, inflammation, hepatic dysfunction, biliary obstruction, and treatment toxicity, and no single instrument captures all of these domains. PG-SGA, GLIM, and CONUT were designed for different purposes—symptom-guided assessment and intervention planning, criteria-based diagnosis with severity grading, and laboratory-based prognostic risk stratification—and the available HPB evidence, although limited and largely retrospective, shows that they identify different patients and carry different confounders.

The prevalence and prognostic value of GLIM depend on how its muscle mass criterion is operationalized, for which staging CT can be used. PG-SGA is the only one of the three designed to link assessment directly to intervention, and CONUT should be interpreted as a marker of combined nutritional, inflammatory, and hepatic compromise rather than as a diagnosis of malnutrition, particularly in cirrhosis and obstructive jaundice. Because direct head-to-head evidence in HPB cancer remains limited, none of the three tools can be considered superior to the others. Tool selection should therefore follow the clinical question, tumor type, treatment phase, and available resources. The framework proposed here is intended to structure that choice and to define the comparisons that prospective, HPB-specific studies should now test, including whether tool-guided nutritional intervention improves outcomes.

## Figures and Tables

**Table 1:** Nutritional instruments discussed in this review, by design purpose.

Instrument	Class/Inputs	Designed Purpose	Role in This Review
NRS-2002, MST, MUST	Screening questionnaires (recent weight loss, intake, BMI, disease severity)	Screening: identify patients at risk who need assessment	Comparators; screening step of the GLIM sequence
SGA/PG-SGA	Clinician (and patient)-completed history and physical examination; scored	Assessment: characterize nutritional deterioration and plan intervention	Index tool (PG-SGA); SGA as comparator
GLIM	Phenotypic + etiologic criteria after a positive screen	Diagnosis: criteria-based malnutrition with severity grade	Index tool
CONUT	Serum albumin, total cholesterol, total lymphocyte count	Prognostic risk stratification (nutritional–inflammatory)	Index tool
PNI, GNRI, CRP–albumin ratio	Albumin-based laboratory indices	Prognostic risk stratification	Comparators
CT skeletal-muscle index; bioimpedance phase angle	Direct body composition measurement	Phenotypic input (muscle mass) for GLIM; independent prognostic factor	Comparators; operationalize the GLIM muscle mass criterion

NRS-2002, Nutritional Risk Screening 2002; MST, Malnutrition Screening Tool; MUST, Malnutrition Universal Screening Tool; SGA, Subjective Global Assessment; PG-SGA, Patient-Generated Subjective Global Assessment; GLIM, Global Leadership Initiative on Malnutrition; CONUT, Controlling Nutritional Status; PNI, Prognostic Nutritional Index; GNRI, Geriatric Nutritional Risk Index; CRP, C-reactive protein; CT, computed tomography; BMI, body mass index.

**Table 2:** HPB-specific evidence for PG-SGA, GLIM, and CONUT by tumor type, with evidence classification.

Tumor Type	Tool	Representative Evidence (Design)	Key Finding	Evidence Classification ^1^
** PANCREATIC CANCER**				
	**PG-SGA**	Prospective and retrospective cohorts; systematic review	Category stratifies survival; captures nutrition-impact symptoms and functional decline; survival-prognostic value not confirmed in systematic review	HPB-specific; Level 2–3; prognostic signal unconfirmed [17,18,19]
	**GLIM**	Cross-sectional agreement study; retrospective pancreaticoduodenectomy cohort	Substantial agreement with PG-SGA (κ 0.71–0.81); predicts major morbidity after pancreaticoduodenectomy	HPB-specific; Level 3; consistent across two studies [20,21]
	**CONUT**	Propensity-matched cohorts; meta-analysis	Independently predicts overall survival and outperformed PNI in one cohort; recurrence and complication signals inconsistent and cutoff-dependent (≥3 to ≥5)	HPB-specific; Level 1 for overall survival [22]; Level 3 for recurrence and complications, inconsistent [23,24,25,26,27]
** HEPATOCELLULAR CARCINOMA**				
	**PG-SGA**	Prospective and retrospective cohorts	Predicts 1-year mortality; score ≥4 predicts post-hepatectomy complications; may underdetect CT-defined sarcopenia	HPB-specific; Level 2–3; consistent for short-term outcomes [28,29,30]
	**GLIM**	Single-center retrospective cohort; multicenter nomogram cohort	Prevalence and prognostic value depend on including CT muscle; prognostic with the muscle criterion, non-significant without	HPB-specific; Level 3; consistent direction, sensitive to muscle criterion operationalization [31,32]
	**CONUT**	Resection cohorts; meta-analyses	Prognostic, but correlates with Child–Pugh class and cirrhosis rather than tumor factors; loses independence versus PNI and ALBI; no prognostic value after liver transplantation	HPB-specific; Level 1 [33,34] and Level 3 [35,36,37,38,39,40]; consistently prognostic, independence from liver function scores not established
** CHOLANGIOCARCINOMA/GALLBLADDER CANCER**				
	**PG-SGA**	No dedicated biliary study; mixed HPB surgical cohorts	Robust to cholestatic laboratory distortion in principle; dedicated biliary data absent	Extrapolated; Level 3 indirect support only [41,42]
	**GLIM**	Small single-center BTC cohort	Severe malnutrition predicts survival on adjuvant chemotherapy	HPB-specific; Level 4; single study [43]
	**CONUT/PNI**	Meta-analysis (PNI); retrospective cohorts (CONUT)	High CONUT predicts worse survival but is associated with jaundice; PNI predicts survival; PNI + ALBI outperforms both	HPB-specific; Level 1 for PNI [14]; Level 3 for CONUT [44,45,46,47,48]; consistent but confounded by jaundice

^1^ Evidence classification (see Section 2): directness = HPB-specific (derived from pancreatic, hepatocellular, or biliary cancer cohorts) or extrapolated (derived from general oncology/gastrointestinal data); design level adapted from the OCEBM 2011 [16]. Levels of Evidence for prognostic questions (Level 1: systematic review or meta-analysis of cohort studies; Level 2: prospective (inception) cohort; Level 3: retrospective cohort or control arm of a randomized trial; Level 4: case series, case–control study, or small single-center cohort at high risk of bias.). PSM, propensity-score matching; PNI, Prognostic Nutritional Index; ALBI, albumin–bilirubin; BTC, biliary tract cancer; HCC, hepatocellular carcinoma. Biliary tract and PG-SGA prognosis cells remain the sparsest.

**Table 3:** Comparison of PG-SGA, GLIM, and CONUT in hepatopancreatobiliary cancer.

Dimension	PG-SGA	GLIM	CONUT
**Designed purpose**	Nutritional assessment and intervention planning	Diagnosis of malnutrition with severity grading	Prognostic risk stratification
**Type/construct measured**	Comprehensive clinical assessment of nutritional deterioration (symptoms, intake, weight, function)	Standardized diagnostic framework (phenotypic + etiologic criteria)	Laboratory-based nutritional–inflammatory risk score
**Components/inputs**	Patient-reported weight change, intake, nutrition-impact symptoms, function; clinician exam of muscle, fat, fluid	Phenotypic (weight loss, low BMI, reduced muscle mass) + etiologic (reduced intake/assimilation, inflammation/disease)	Serum albumin + total cholesterol + total lymphocyte count (score 0–12)
**Meaning of an abnormal result**	Symptom- and intake-driven malnutrition; directly interpretable as nutrition	Formal malnutrition diagnosis with severity grade	Combined inflammatory–metabolic–hepatic reserve risk; not specific to malnutrition
**Proportion classified as abnormal in comparative HPB cohorts**	Highest; PG-SGA > GLIM in HPB surgery [41,42]	Intermediate; more specific than PG-SGA [41]	Poor classifier of GLIM-diagnosed malnutrition; specific but insensitive in non-HPB cohorts [95,96]
**Key confounders in HPB disease**	Subjectivity; may underdetect occult sarcopenia in cirrhosis	Dependent on the muscle-mass criterion; ascites/oedema distort weight and BMI; cutoffs vary	Albumin/cholesterol/lymphocytes confounded by inflammation, cholestasis, jaundice, liver dysfunction, infection, chemotherapy
**Feasibility**	Most labor-intensive; trained assessor; no labs or imaging	Intermediate; can incorporate CT body composition (usually available from staging)	Least resource-intensive; three automated labs; no patient contact
**Reproducibility**	Rater- and effort-dependent	Depends on screening tool and muscle input	Rater-free, but cutoffs heterogeneous (≥3 to ≥5)
**Proposed role**	Symptom-guided assessment, intervention planning, longitudinal monitoring	Standardized diagnosis and severity grading	Rapid automated prognostic/risk stratification (adjunct, not diagnosis)

PG-SGA, Patient-Generated Subjective Global Assessment; GLIM, Global Leadership Initiative on Malnutrition; CONUT, Controlling Nutritional Status; BMI, body mass index; HPB, hepatopancreatobiliary. The three tools measure overlapping but distinct constructs and are complementary rather than interchangeable.

## Data Availability

No new data were created or analyzed in this study. Data sharing is not applicable to this article.
